# Supramolecular Bait to Trigger Non‐Equilibrium Co‐Assembly and Clearance of Aβ42

**DOI:** 10.1002/anie.202013754

**Published:** 2020-12-27

**Authors:** Te‐Haw Wu, Rai‐Hua Lai, Chun‐Nien Yao, Jyh‐Lyh Juang, Shu‐Yi Lin

**Affiliations:** ^1^ Institute of Biomedical Engineering and Nanomedicine National Health Research Institutes (NHRI) Miaoli County 35053 Taiwan; ^2^ Institute of Molecular and Genomic Medicine NHRI Taiwan

**Keywords:** amyloid *β*-peptides, polysaccharides, proteins, non-equilibrium processes, supramolecular chemistry

## Abstract

In living systems, non‐equilibrium states that control the assembly‐disassembly of cellular components underlie the gradual complexification of life, whereas in nonliving systems, most molecules follow the laws of thermodynamic equilibrium to sustain dynamic consistency. Little is known about the roles of non‐equilibrium states of interactions between supramolecules in living systems. Here, a non‐equilibrium state of interaction between supramolecular lipopolysaccharide (LPS) and Aβ42, an aggregate‐prone protein that causes Alzheimer's disease (AD), was identified. Structurally, Aβ42 presents a specific groove that is recognized by the amphiphilicity of LPS bait in a non‐equilibrium manner. Functionally, the transient complex elicits a cellular response to clear extracellular Aβ42 deposits in neuronal cells. Since the impaired clearance of toxic Aβ42 deposits correlates with AD pathology, the non‐equilibrium LPS and Aβ42 could represent a useful target for developing AD therapeutics.

## Graphical abstract

Here, a non‐equilibrium state of interaction between supramolecular lipopolysaccharide (LPS) and Aβ42, an aggregate‐prone protein that causes Alzheimer's disease (AD), was identified. Oscillation of the non‐equilibrium state for co‐assembly and clearance of Aβ42 is triggered through the use of a supramolecular bait. Since the impaired clearance of toxic Aβ42 deposits correlates with AD pathology, the LPS and Aβ42 non‐equilibrium could represent a useful target for developing AD therapeutics.

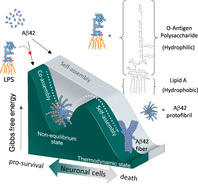

Non‐equilibrium states,[^1^, ^2^, ^3^] or more specifically, far‐from‐equilibrium conditions have been found to be critical for the modulation of biomolecular assembly in living systems.^[4]^ One interesting example is the role of microtubules in the formation of cytoskeleton networks, an assembly process that exhibits a non‐equilibrium behavior for its self‐assembly and infrequent decay.^[5]^ Another example is the amyloid peptides (Aβ), which undergo self‐aggregation to form amyloid plaques in triggering the neurodegenerative cascade of Alzheimer's disease (AD).[^6^, ^7^, ^8^] Among the various Aβ isoforms, Aβ40 and Aβ42 are the two most abundant species. Although Aβ40 is more abundant than Aβ42 in cerebrospinal fluid, Aβ42 has a higher self‐aggregation potential than Aβ40 in contributing to the amyloid deposits in AD brains.^[9]^ Intriguingly, in addition to its pathological role in AD, Aβ42 has recently been implicated in antimicrobial activity against bacterial infection.[^10^, ^11^, ^12^] The self‐propagating Aβ42 may kill bacteria by agglutinating the polysaccharide of bacterial outer membranes.^[13]^ From the perspective of chemical kinetics, the self‐propagation of Aβ42 represents a dynamic self‐assembly process involving a transition state from the pre‐organized protofibrils to a stable form of fibrils.[^14^, ^15^, ^16^, ^17^, ^18^, ^19^] On the other hand, LPS acts as an amphiphilic supramolecule with characteristics of spontaneous self‐assembly in a concentration‐dependent manner when dispersed in solution.^[20]^ One recent intriguing feature identified in AD brains is the co‐localization of bacterial debris (i.e., LPS) with Aβ42 deposits.^[21]^ The large amounts of LPS in the brain might elicit inflammatory responses in promoting AD development.^[22]^ However, one cannot exclude the possibility that there could be a physiological or protective function that has not been defined in normal or early AD brains. Inspirited by this, we proposed that these two supramolecules might form an intermolecular interaction in a non‐equilibrium state that could subsequently impact on the survival of neural cells (Figure 1).

**Figure 1:**
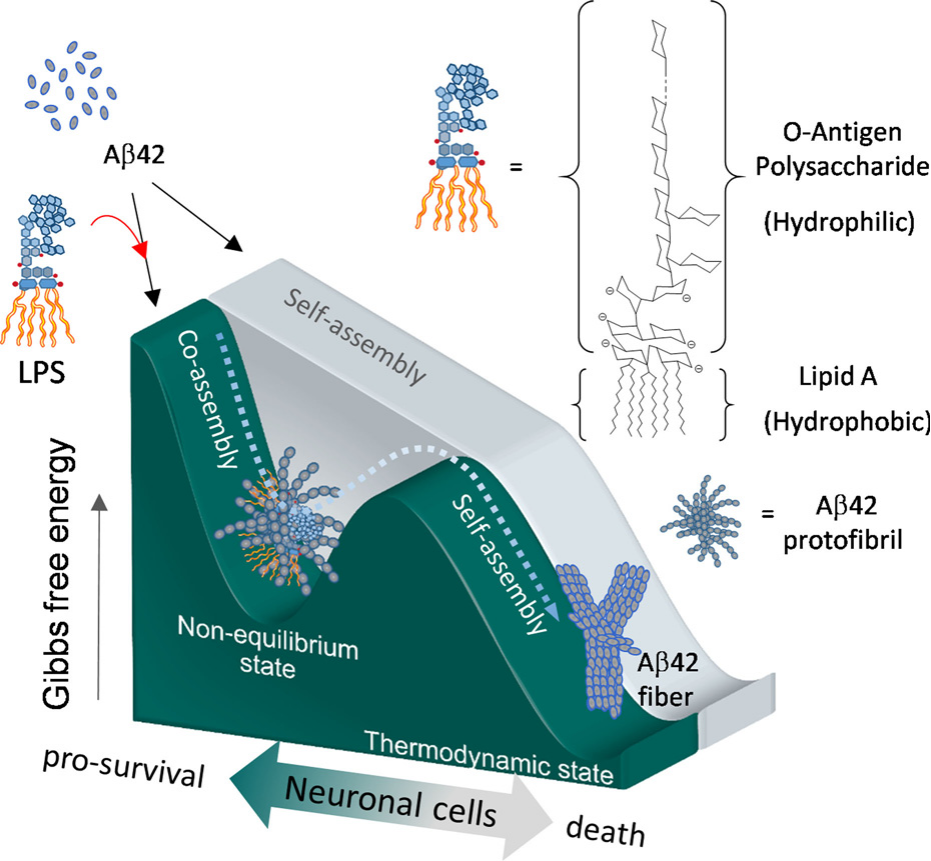
A hypothesized model describing that LPS might behave a supramolecular bait to trigger non‐equilibrium co‐assembly with Aβ42 protofibrils for pro‐survival effect of neuronal cells.

To test this hypothesis, we investigated whether LPS could form a transient complex with Aβ42 through a non‐equilibrium co‐assembly process that subsequently leads to dissociation. Bis‐ANS, a specific fluorescent dye sensitive to Aβ42 hydrophobicity,^[23]^ was used to assess the degree of Aβ42 hydrophobicity during the process of amyloid polymerization upon the administration of LPS. The results revealed a repeated oscillating pattern of association and dissociation between the two molecules (Figure 2 a). Specifically, the Aβ42 hydrophobicity was noticeably increased and then rapidly reduced back to a baseline level within a 30 min incubation period after adding fresh LPS. This result suggested that the Aβ42 protofibrils might induce transient LPS‐Aβ42 binding when they first encounter LPS in solution (Figure 2 b). After the rapid growth of hydrophobicity, the continuing oscillation became well dampened if freshly prepared LPS was re‐administered, with more pronounced effects being noted if LPS of higher concentration was re‐administered. Similar effects were noted if an aged LPS (LPS being self‐incubated overnight) was re‐administered, but the dampening of Aβ42 hydrophobicity became small. Since LPS is also an amphiphilic supramolecule that favors self‐aggregation,^[24]^ we reasoned that aged LPS might become less competent than fresh LPS in forming LPS‐Aβ42 complex. Despite the amyloid fibrils being found to continuously form in the presence of LPS over the prolonged incubation time, the formation of long fibers of Aβ42 was clearly weakened (see Figure S1 in the Supporting Information). These results suggest a dissipative non‐equilibrium state of self‐aggregation for Aβ42 might be induced by LPS.

**Figure 2:**
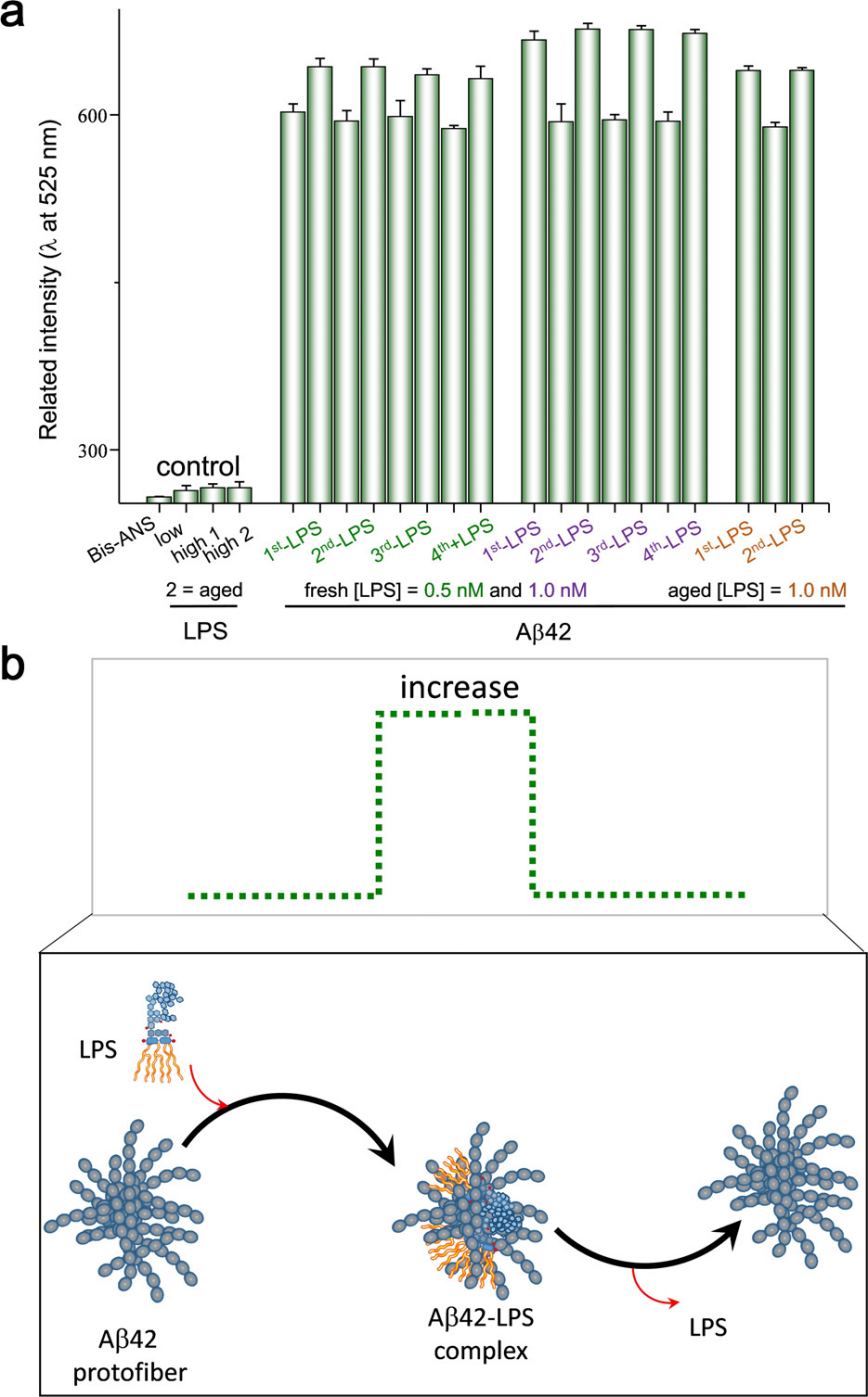
Confirmation of non‐equilibrium interaction between LPS and Aβ42 protofibrils. a) Oscillation of the hydrophobicity of Aβ42 was noted by repeatedly adding freshly prepared LPS or aged LPS. The *y*‐axis of the graph represents the relative fluorescence intensity of the bis‐ANS emission wavelength at 525 nm. b) A simple illustration showing the hydrophobicity of Aβ42 protofibrils could increase and decrease by LPS influx and efflux, respectively.

Next, we investigated whether the transient association between LPS and Aβ42 protofibrils impacts cytotoxicity. Surprisingly, cell viability assays suggested that SH‐SY5Y neuronal cells retained more than 95 % cell viability after co‐treatment of Aβ42 with LPS. In contrast, less than 20 % cell viability was retained in cells treated with Aβ42 alone (Figure S2, columns 2 and 3). To evaluate if the rescue effect was mediated through the LPS‐Aβ42 transient complex, we removed the unbound LPS from the solution and found that the rescue effect of LPS was completely lost (Figure S2, columns 3 and 4). However, when LPS were re‐added back into the medium, the cell viability was returned to 85 % of the normal value in control cells (Figure S2, columns 1 and 5). Moreover, the rescue effect of LPS on the Aβ42‐induced apoptosis was found to occur in a dose‐dependent manner with increasing LPS (Figure 3 a). That being the case, how is this accomplished? Under normal conditions, both the oligomeric (protofibrils) and the monomeric forms of Aβ42 can be internalized from the extracellular domains of brain cells for degradation.[^25^, ^26^] However, during the pathogenesis of AD, the process of Aβ42 protofibril clearance via the endocytic pathway is interrupted, leading to an increased deposition of Aβ42.[^27^, ^28^] Accordingly, we speculated that the LPS‐Aβ42 complex might restore the endocytic clearance of the Aβ42 peptides. To test this, we set out to investigate whether the Aβ42 levels and aggregation were both decreased. Western blot analysis of Aβ42 peptides collected from the entire volume of the cell culture medium and total cell lysates suggested that the extracellular and intracellular proteins of Aβ42 were markedly depleted upon co‐treatment with LPS (Figure 3 b, lanes 3–5). However, in the absence of cells, the Aβ42 levels and oligomeric states were remained unchanged by the LPS (Figure 3 b, lanes 2 and 6), suggesting that the formation of transient complex between LPS and Aβ42 triggered a potent cellular response that resulted in the degradation of the Aβ42 peptides. These data provide strong evidence to support a role for the non‐equilibrium complex of Aβ42‐LPS in rescuing cells from death through promoting the clearance of Aβ42 protofibrils.

**Figure 3:**
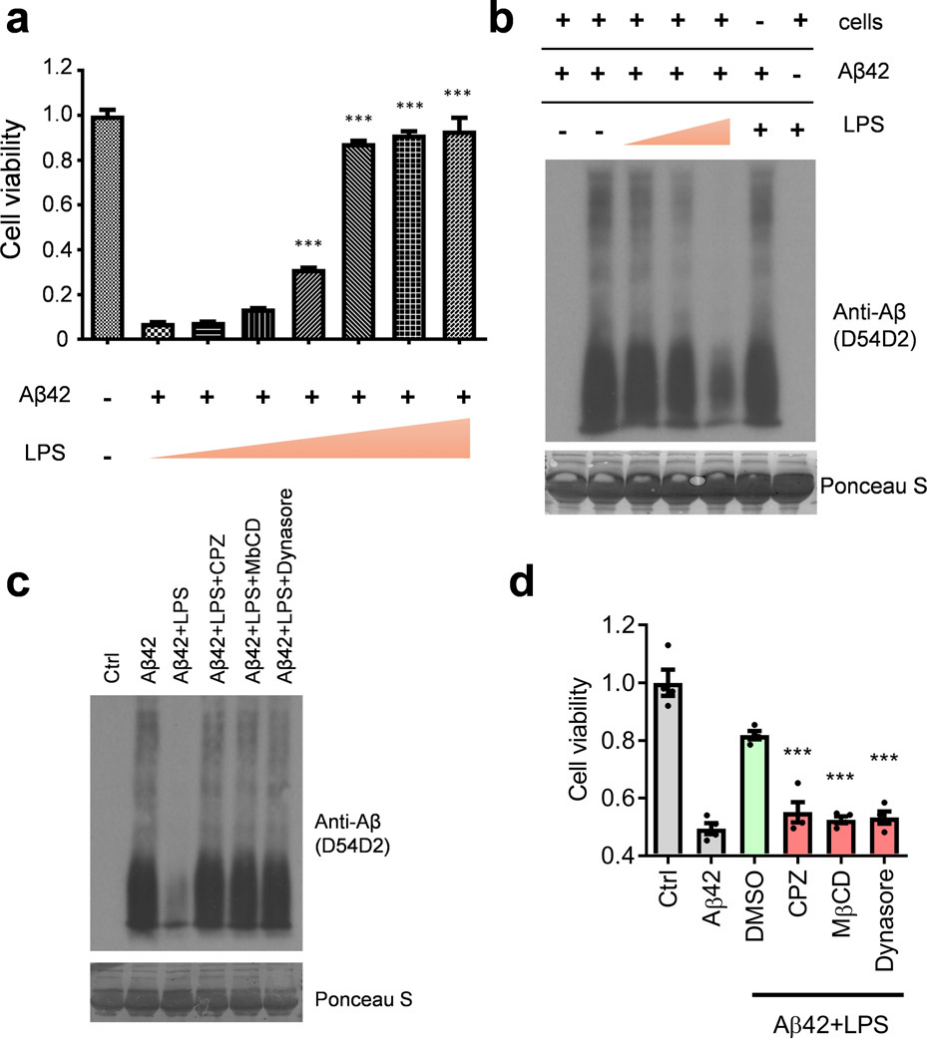
Aβ42 peptides and neuronal toxicity are diminished in neuronal cells co‐treated with LPS. a) The reduced cell viability effect of Aβ42 on SH‐SY5Y cells is significantly improved by co‐treatment with LPS in a dose‐dependent manner. b) Western blotting indicating Aβ42 degradation in cells co‐treated with or without LPS. c) The LPS‐induced degradation of Aβ42 peptides is suppressed by three endocytosis blockers: chlorpromazine (CPZ), methyl‐β‐cyclodextrin (MβCD), and dynasore. d) The rescue effect of LPS against Aβ42 neuronal toxicity is blocked by endocytosis inhibitors. ***, *P*<0.001.

To understand the underlying mechanisms, we first tested whether matrix metalloproteinases (MMPs) secreted from cultured cells might mediate the destruction of Aβ42 peptides in the extracellular milieu. However, the treatment of neuronal cells with pan‐MMP inhibitors showed no changes in Aβ42 levels in cells co‐treated with LPS (Figure S3). Accordingly, we turned our attention to the intracellular protein degradation pathways. We used pharmacological blockers to inhibit endocytosis mediated by the clathrin‐ and dynamin‐dependent mechanisms. The results suggested that the blockade of the endocytic uptake of extracellular Aβ42 effectively abolished the amyloid degradation (Figure 3 c, lanes 4–6) as well as the pro‐survival effect of LPS rescuing SH‐SY5Y cells from the Aβ42‐induced apoptosis (Figure 3 d). Since autophagy is also a known cellular mechanism for endolysosomal degradation,^[29]^ we inhibited autophagy with two typical inhibitors, methyladenine (3MA) and bafilomycin A1 (BA), and found very comparable effects to those observed for the endocytosis blockers (Figure S4). The results strongly suggest that an endolysosomal pathway mediates the degradation of Aβ42 peptides in neuronal cells co‐treated with LPS.

Furthermore, it is essential to clarify (i) why LPS can only bind with Aβ42 but not with Aβ40 (Figure S5) and (ii) how they bind together. To understand the binding preference, we speculated that Aβ42 might be more prone to fast fibrillization that possibly constructs surfactant properties to increases the LPS‐Aβ42 interaction in comparison to the interaction with Aβ40. As expected, Aβ42 was found to still undergo rapid fibrillization in both the presence and absence of LPS (Figure S6), a phenomenon that was not observed for Aβ40. Additionally, small‐angle X‐ray scattering analysis showed that Aβ42 increased the critical aggregation concentration (CAC) of LPS from 3.49±0.052 μg mL^−1[30]^ to 15.91±0.13 μg mL^−1^ (Figure S7, left panel), whereas the CAC of LPS was found to not be obviously affected by the presence of Aβ40 (3.82±0.07 μg mL^−1^, Figure S7, right panel). The increased CAC of LPS by Aβ42 suggested that a mutual interaction between LPS and soluble Aβ42 protofibrils occurs to suppress the self‐assembly process of LPS.

Prompted by the finding that Aβ42 formed a complex with amphiphilic LPS, we speculated that the soluble Aβ42 protofibrils might possess a specific groove that acts as a kinetic trap and enables the docking of amphiphilic LPS. To test this possibility, we used a unique LPS sequester, an atomic sheetlike gold nanocluster (identified as SAuM) with a specific dock for the lipid A of the hydrophobic domain, that has been demonstrated in our previous work.[^30^, ^31^] Indeed, the binding efficiency of Aβ42 protofibrils and LPS was found to be significantly decreased in the presence of the SAuM (Figure 4 a, black line). Furthermore, we also showed that colistin, a cyclic peptide which is known to cap the hydrophilic domain (O‐antigen) of LPS,^[32]^ exerted a similar effect in decreasing the binding efficiency of Aβ42 protofibrils to LPS (Figure 4 a, red line). Both results indicated that the Aβ42 protofibrils possess LPS‐specific amphiphilic grooves that allow docking with the hydrophobic and hydrophilic domains of LPS. Accordingly, we abolished the complex formation of LPS and Aβ42 by blocking the lipid A or O‐antigen binding sites in LPS with the above two inhibitors. The results clearly showed that the roles of LPS in promoting the clearance of Aβ42 protofibrils (Figure 4 b) or in attenuating the cytotoxicity of Aβ42 were both compromised (Figure S8). We then integrated these results into a model of the structural–functional interaction between LPS and Aβ42 in modulating the endocytic clearance of Aβ42 in neuronal cells (Figure 4 c).

**Figure 4:**
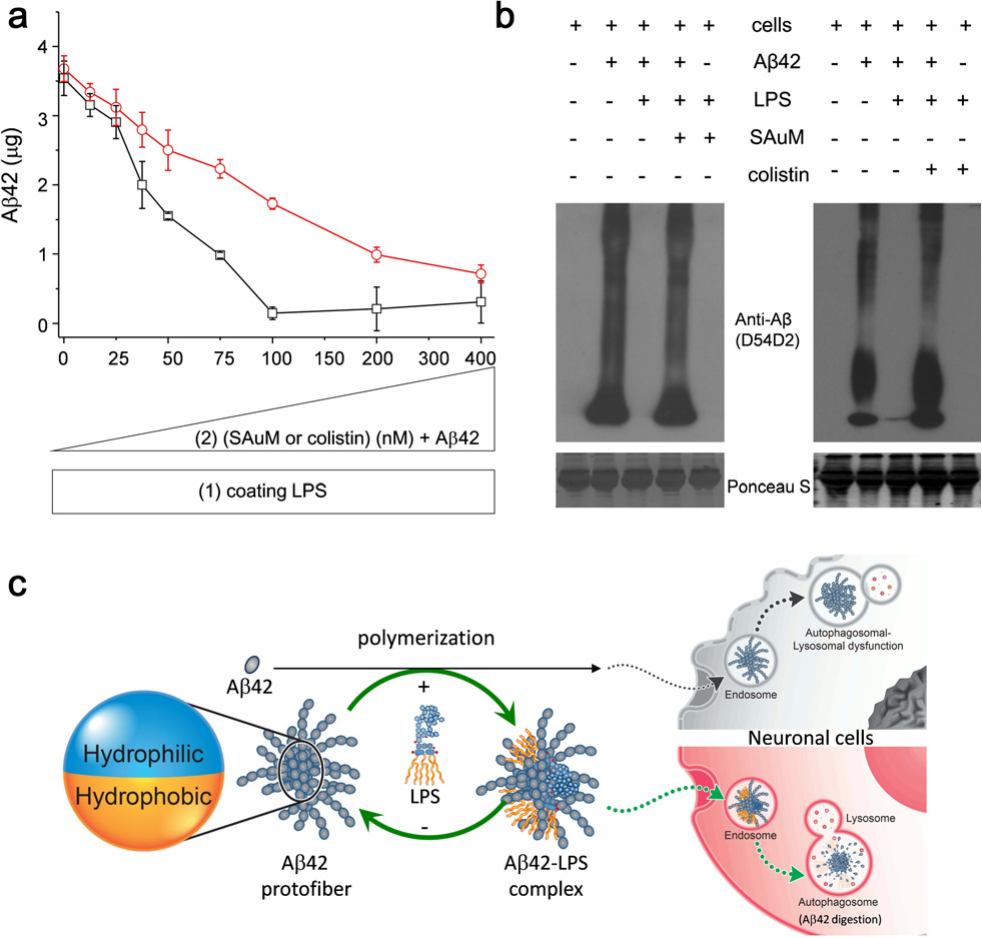
The amphiphilic groove of Aβ42 protofibrils is required for the binding of LPS and the induction of neuronal clearance of Aβ42. a) Two decay curves revealing that the complex formation of Aβ42 protofibrils and LPS was impeded in the presence of antagonistic O‐antigen (red circles, as colistin blocked the hydrophilic domain of LPS) or lipid A (black squares, as SAuM blocked the hydrophobic domain of LPS). b) LPS antagonists effectively suppressed the Aβ42 degradation induced by LPS in cells. c) Pictorial illustration showing that LPS may induce an endo‐lysosomal clearance of Aβ42 in neuronal cells via the formation of a non‐equilibrium complex with the Aβ42 protofibrils.

In conclusion, we demonstrated that the specific groove of Aβ42 protofibrils is important for complexing with amphiphilic LPS through a pattern of non‐equilibrium behavior. We also speculate that LPS may actually act as a bait in attracting the Aβ42 intermediates that sometimes deviate from their supposed thermodynamic self‐assembly process. With respect to functionality, we provide evidence supporting the conclusion that such a transient supramolecule–supramolecule interaction can potently stimulate a strong cellular response toward autophagy‐mediated protein degradation for Aβ42 peptides in neuronal cells. Moreover, since the oscillation of the non‐equilibrium state appears to be sustainably maintained, the extracellular Aβ42 protofibrils were eventually diminished over a prolonged incubation time with the cells. Altered homeostasis between Aβ peptide production and clearance is defined as the pathological basis for the accumulated Aβ fibrils in AD brains. Since efforts aimed at blocking Aβ42 production have not been successful, the current strategies of AD drug design have been shifted to target Aβ clearance and degradation. The current work is important because it provides a new strategy for structure‐based drug design to discover therapeutics that promotes Aβ42 clearance.

## Conflict of interest

The authors declare no conflict of interest.

## Supporting information

As a service to our authors and readers, this journal provides supporting information supplied by the authors. Such materials are peer reviewed and may be re‐organized for online delivery, but are not copy‐edited or typeset. Technical support issues arising from supporting information (other than missing files) should be addressed to the authors.

Supplementary
