# Supplementary material for: Supramolecular Bait to Trigger Non‐Equilibrium Co‐Assembly and Clearance of Aβ42
Source: Angew Chem Int Ed Engl. 2020 Dec 27;60(8):4014–7. doi: 10.1002/anie.202013754 (PMC7898541; doi:10.1002/anie.202013754)
Supplement: Supplementary file 1 — Supplementary [file ANIE-60-4014-s001.pdf]

Angewandte Chemie
Eine Zeitschrift der Gesellschaft Deutscher Chemiker

Supporting Information

Supramolecular Bait to Trigger Non-Equilibrium Co-Assembly and Clearance of Aβ42

Te-Haw Wu[+], Rai-Hua Lai[+], Chun-Nien Yao[+], Jyh-Lyh Juang, and Shu-Yi Lin**

anie_202013754_sm_miscellaneous_information.pdf

1. Materials and Methods
2. Figure S1-S8
3. Supplementary reference list

Materials and Methods

An LPS-coated plate for Aβ42 and Aβ40 binding assay. 100 mM Na_2CO_3, 20 mM EDTA, and 0.1 mL of 30 μg/mL LPS solution were added to a 96-well immunoassay plate (Costar 9018, Corning Corporation), which was then incubated at 37 °C for 3 hours. The coated plate was then washed with deionized water and dried for one day. PBS solution containing 1% BSA was then added to block the coated plate at 37 °C for 30 minutes. Finally, the coated plate was washed three times with PBS solution containing 0.1% BSA. Subsequently, different concentrations of Aβ42 protofibrils (with or without added SAuM or colistin) were added to the coated wells and incubated at 37 °C for 16 hours, before being washed three times with PBS. Bio-red protein staining dye was then added into the coated wells to assay the Aβ42 or Aβ40 concentrations of each well and measure the absorption value at 625 nm using an ELISA reader. The values were substituted into the calibration curve to calculate the concentrations of Aβ42 or Aβ40 in each well.

Polymerization rate of Aβ42. The methods for the liquid type were similar to those used for the LPS-coated plate, but a filter was used to remove Aβ42 fibers during the sample preparation. In brief, a mixture of Aβ42 and LPS was incubated at 37 °C in a time-dependent manner. Then, the Aβ42 fibers were removed using a 0.1-um filter of MWCO (Millipore MILLEX-HP) to avoid interference. The Aβ42 filtrate was collected and dried before adding bio-red protein staining dye, and the absorption value of the filtrate was then measured at 625 nm using an ELISA reader. The values were substituted into the calibration curve to calculate the concentrations of Aβ42 in each well.

Identification of the non-equilibrium steady state between LPS and Aβ42. A RPMI medium containing Aβ42 (100 µM) and LPS (1.0 nM) was incubated at 37 ^{o}C. At different time points, a small proportion of the solution was taken out to stain with a fluorescent dye, bis-ANS (1.0 µM, 4,4′-Dianilino-1,1′-binaphthyl-5,5′-disulfonic acid dipotassium salt, Sigma-Aldrich), and was immediately measured using a fluorescence spectrophotometer (Varian, Cary Eclipse, excitation wavelength at 390 nm).

Transmission electron microscopy (TEM). The mixtures of LPS and individual Aβ42 were prepared in deionized water. Samples were mounted on a 400-mesh Cu grid with carbon supporting film and stained with 2% phosphotungstic acid. Excess staining reagent was removed using a filter paper, and the grid was dried prior to transmission electron microscopy measurements (Hitachi H-7650, Japan) at 100 kV.

Cell viability. Neural cells were maintained in MEM (Gibco 11095-080) supplemented with 10% FBS and 1X MEM NEAA (Gibco 11140-050). Neural SH-SY5Y cells were plated at a density of 8000 cells per well in 96-well plates (Costar 3599) and allowed to attach overnight at 37 °C in 5% CO_2 humidified air. Aβ42 (100 µM), LPS (1000 nM), SAuM (1000 nM), or colistin (1000 nM) were then added to each group medium containing 2.5% dimethyl sulfoxide (DMSO) and sterilized by using UV light. The medium was then replaced every day and the remaining mixture was incubated for 72 hours at 37 °C in 5% CO_2 humidified air. Cell viability was measured using the CCK8 (Sigma-Aldrich 96992) assay according to the manufacturer’s protocol. A 0.22-µm filter (non-pyogenic, Millex®-GV) was used to remove unbound LPS, if needed, in the LPS-Aβ42 solution.

Western Blots. To assess the level of Aβ42 in the cell culture medium, Aβ42 peptides were collected from the entire volume of the cell culture medium and total cell lysates and mixed with protein sample buffer (final 0.1 M Tris-HCl, pH6.8, 10% glycerol, 2% SDS, 1% β-mercaptoethanol, and 0.01% bromophenol blue) for Western blot analysis. The samples of cell mixture were analyzed by SDS-PAGE gel electrophoresis and transferred to a PVDF membrane (Millipore). After blocking with blocking buffer containing 5% w/v nonfat dry milk in PBST (1X phosphate-buffered saline and 0.1% Tween 20) at room temperature for 1 h, the membrane was incubated with a primary antibody diluted in the blocking buffer at 4°C overnight. After hybridization with primary antibody, the membrane was washed three times with PBST before the addition of an HRP-conjugated secondary antibody against the primary antibody. The membrane was then washed three times with PBST before immunoreactive bands were detected by chemiluminescence (PerkinElmer) or Ponceau S staining (Bersting Technology). Primary antibodies used in this study included the following: anti-Aβ (#8243, Cell Signaling Technology), anti-LC3 (#4108, Cell Signaling Technology), anti-cathepsin B (sc-13985, Santa Cruz Biotech), anti-cathepsin D (sc-6486, Santa Cruz Biotech), and anti-GAPDH (GTX100118, GeneTex). Culture medium containing 2.5% dimethyl sulfoxide (DMSO) was used for each group.

Statistical analyses. For biologic assays, we used GraphPad Prism (v7.02) to perform one-way ANOVAs. All data were expressed as mean ±SEM, and P-values of less than 0.05 were considered to be statistically significant and showed asterisks (**, $P<0.01$; ***, $P<0.001$).

The measurement of critical aggregation concentration (CAC). The CAC of LPS was measured by small-angle X-ray scattering (SAXS). The SAXS data for the sample solutions, which were collected using a Pilatus 1M-F detector, were used to extract the zero-angle intensity I_o (q = 0) and radius of gyration R_g of the LPS micelles on the basis of Guinier approximation. The value of CAC was then extracted from the intercept of the linear regression fitting of the concentration-dependent zero-angle intensity I_o (q = 0), as shown in Figure S4.

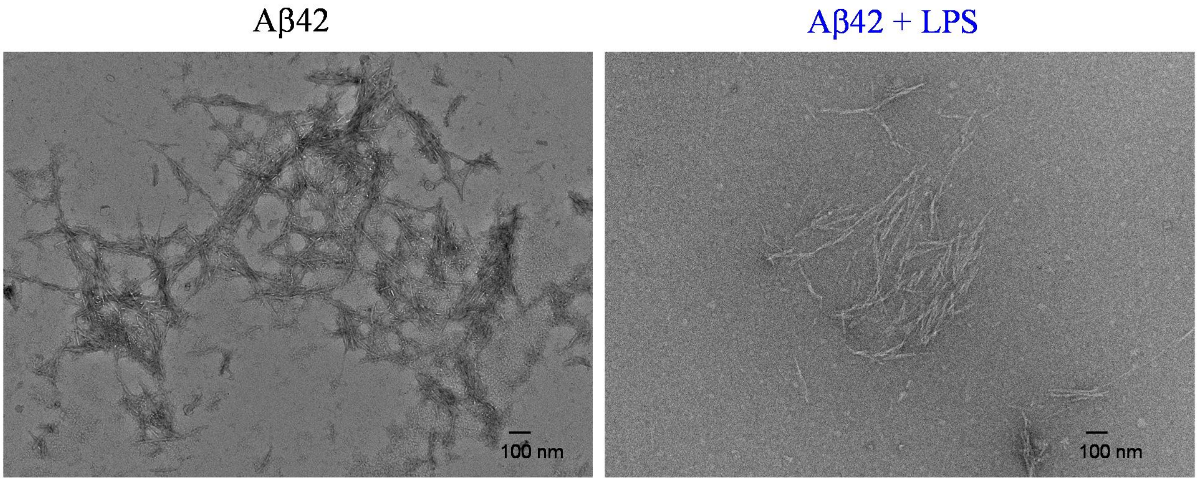

Figure S1. Representative TEM images of insoluble shorter fibers of Aβ42 were noted after co-incubation with LPS.

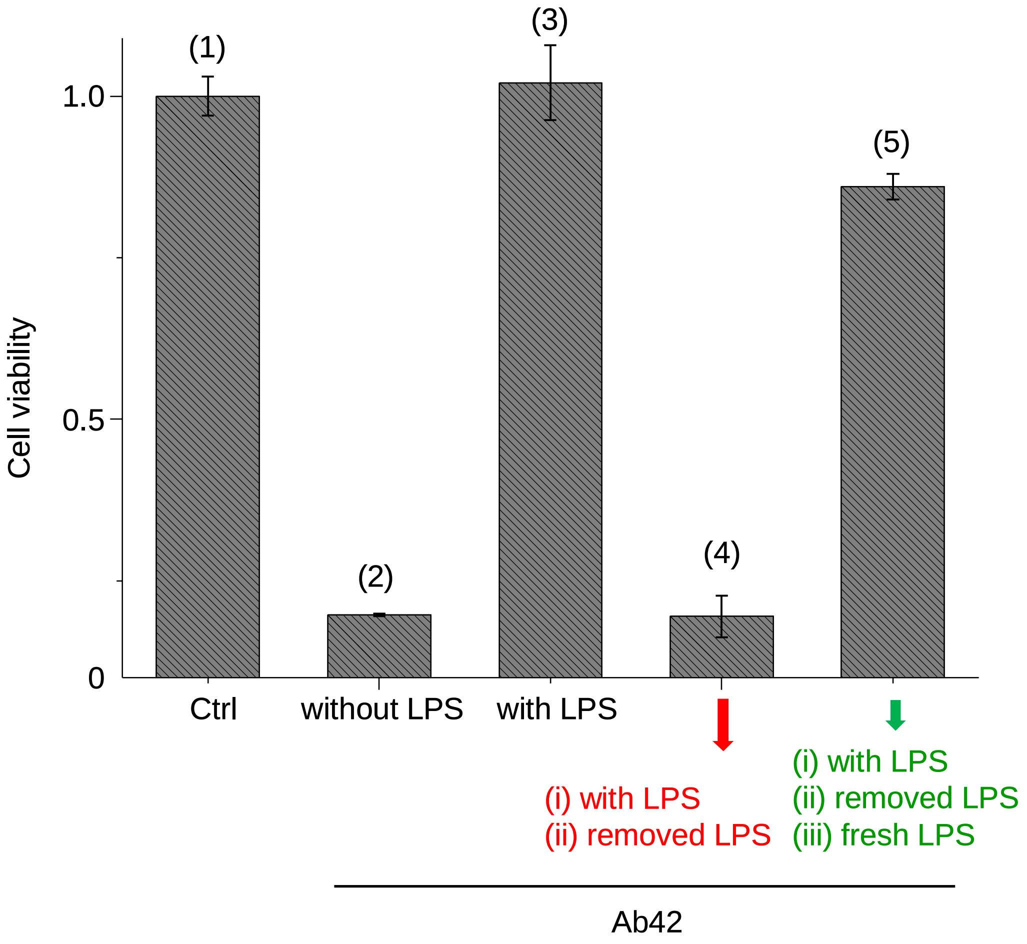

Figure S2. The transient LPS-Aβ42 binding ameliorates the Aβ42-induced neuronal toxicity. The cytotoxic effect of Aβ42 on SH-SY5Y cells was found to be greatly decreased by the co-treatment of Aβ42 with LPS (comparing columns 2 and 3), but that effect was found to be completely abolished by removing the unbound LPS from the solution (comparing columns 3 and 4), whereas adding the LPS back into the solution restored the lost effect (comparing columns 4 and 5). Ctrl represents SH-SY5Y cells only.

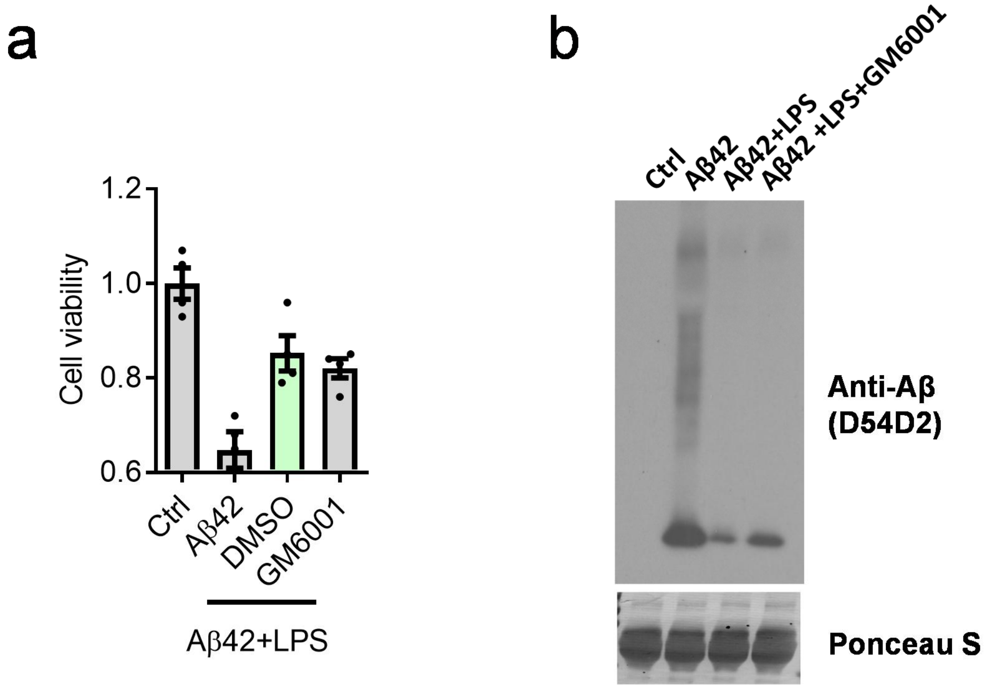

Figure S3. Matrix metalloproteinases do not contribute to the LPS-induced Aβ clearance. (a) The rescue effect of LPS against Aβ42 neuronal toxicity can be observed through the treatment of the neuronal cells with pan-MMP inhibitors. (b) Western blotting of Aβ42 degradation in SH-SY5Y cells in the presence of LPS co-treated with or without pan-MMP inhibitor.

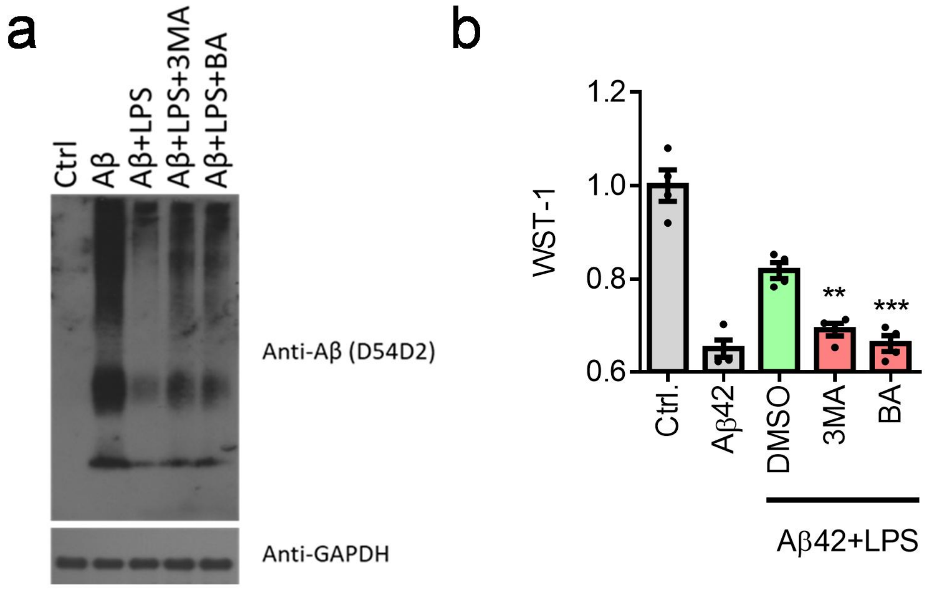

Figure S4. LPS enhances autophagy-lysosome pathway activity in neuronal cells. The co-incubation of LPS-binding Aβ42 complex and either 3MA or BA can cause a cascade of adverse consequences in neural cells: (a) decreasing the degradation of Aβ42 in neural cells; and (b) increasing the cell death of neural cells. Note that "Aβ42" is abbreviated as "Aβ" in (a) and (b). **, $P<0.01$; ***, $P<0.001$.

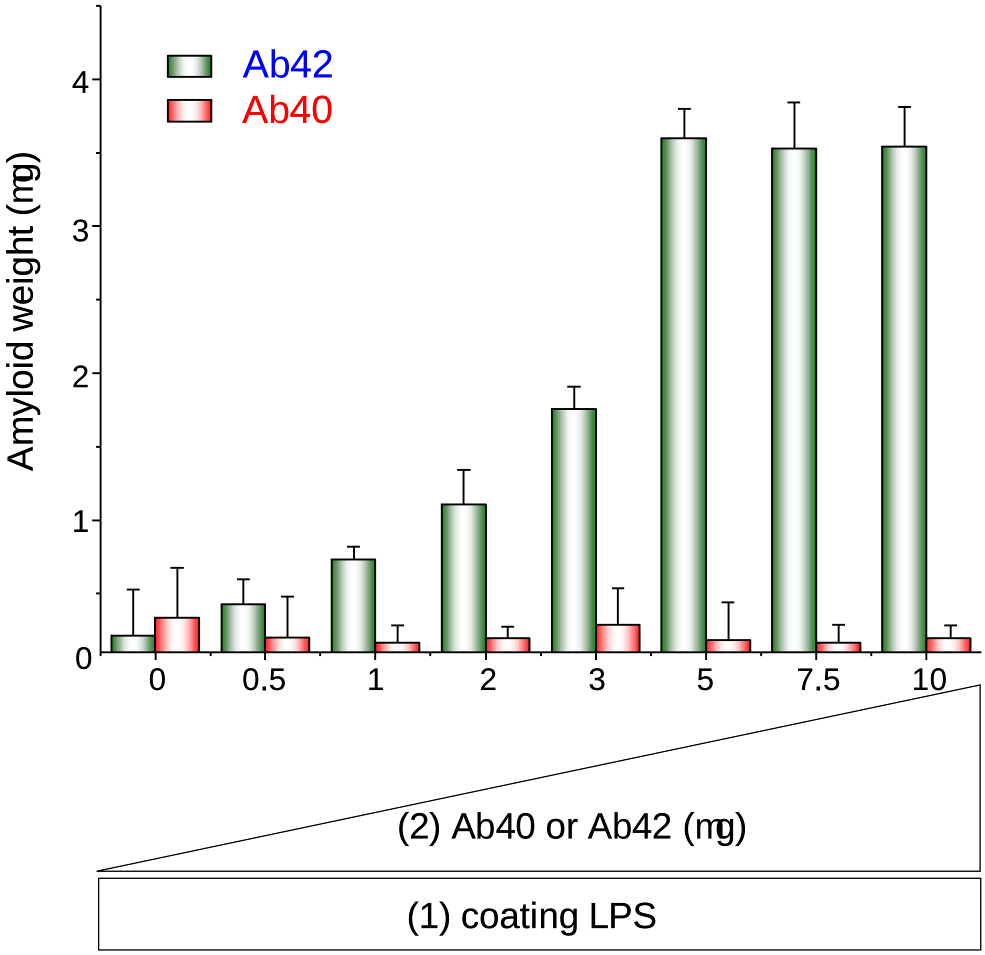

Figure S5. Binding selectivity with LPS was identified from Aβ42, not from Aβ40. Binding assays revealed that the adhesion weight was increased in a dose-dependent manner until a plateau was reached when 0.5-10 mg of Aβ42, but not Aβ40, were added to an LPS-coated plate. The binding percentage of Aβ42 to LPS was approximately 75%.

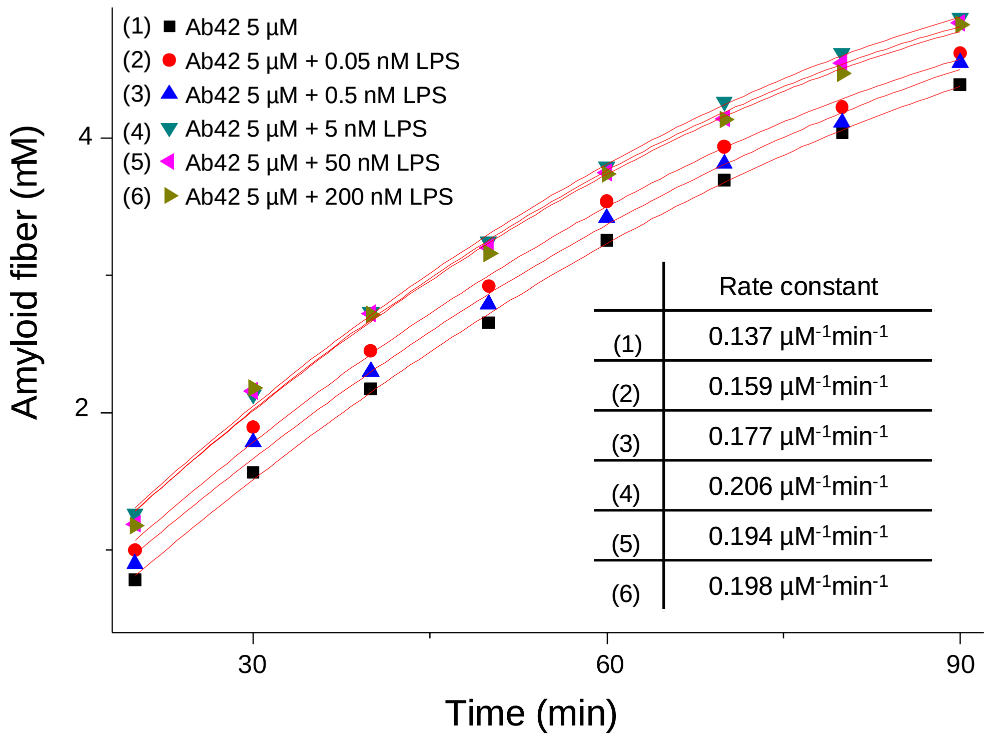

Figure S6. The rate constant of Aβ42 fibrillization in the absence or presence of LPS could be measured in a time-dependent manner through Aβ42 filtrate collection. After adding bio-red protein staining dye, the absorption value of the filtrate was then measured at 625 nm using an ELISA reader. The values were substituted into the calibration curve to calculate the concentrations of Aβ42 in each well. We also found that the rate constant of fibrillization showed a slight change from 0.137 mM^{-1}min^{-1} to 0.198 mM^{-1}min^{-1} (inset table) in a LPS-dependent manner. The finding suggested that the self-propagating rate in the presence of LPS is slightly slower than Aβ42 alone.

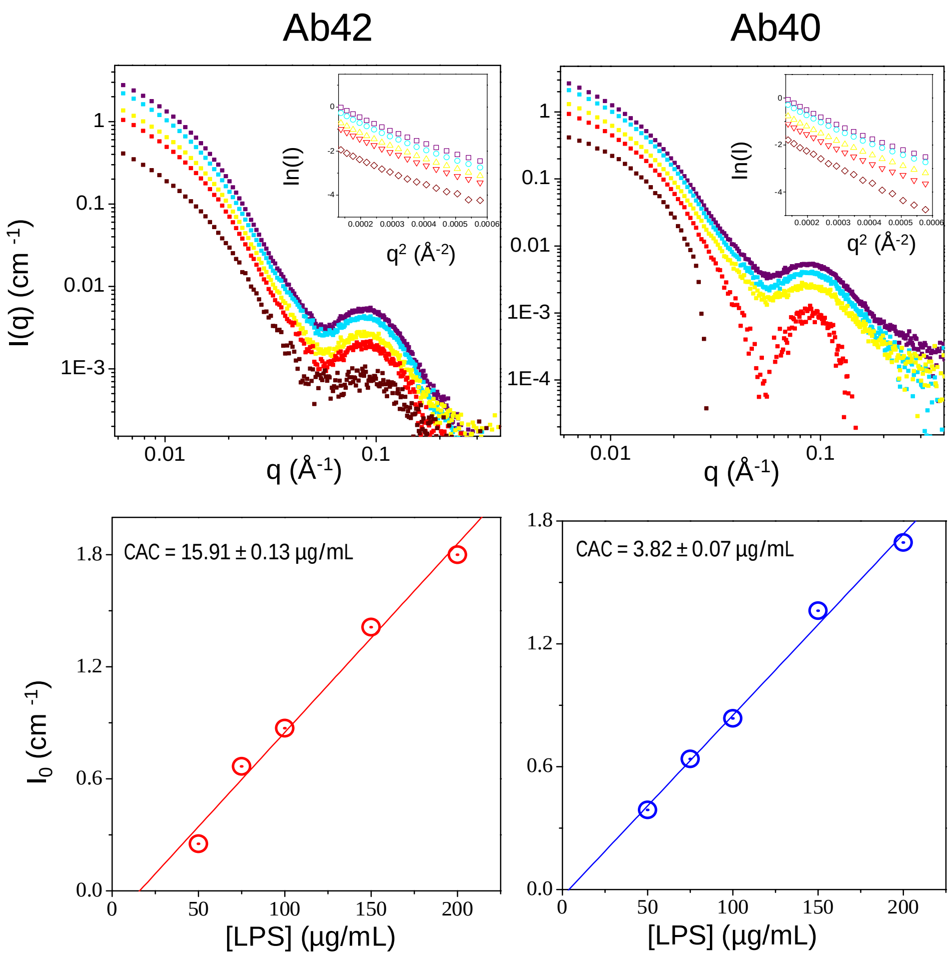

Figure S7. The critical aggregation concentration (CAC) of LPS in the presence of either Ab42 or Ab40 was measured by SAXS. The upper figure shows the plots of scattering intensities as a function of q, defined by q = 4pl $^{-1}$ sin(q) with the scattering angle 2q and X-ray wavelength l . The inset figures show these signals for nascent LPS aggregates at different concentrations in the presence of Aβ42 or Aβ40. The CAC values of the third row of figures include uncertainties (standard deviations).

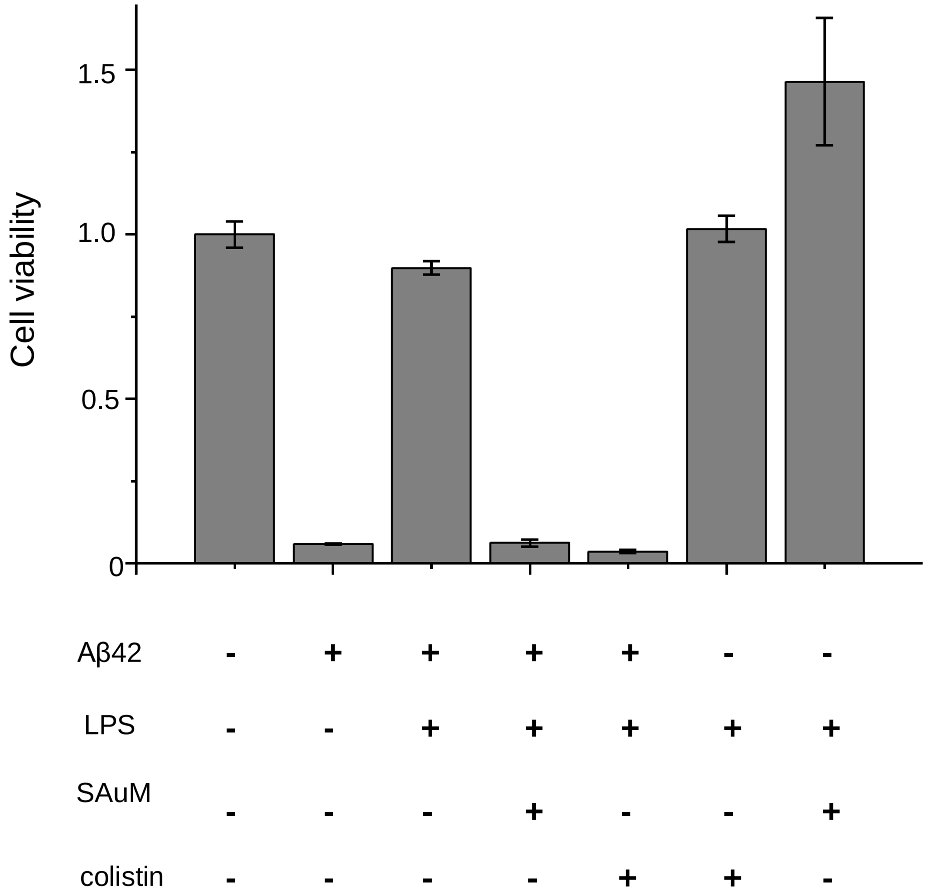

Figure S8. The rescue effect of LPS against Aβ42 neuronal toxicity is blocked by LPS antagonists. The complex formation of Aβ42 protofibrils and LPS resulting in the rescue effect of neural cells was abolished when the O-antigen was antagonized by colistin (a blocker for the hydrophilic domain of LPS) or the lipid A (i.e., the active center of LPS)[1] was antagonized by SAuM (a blocker for the hydrophobic domain of LPS).

Supplementary reference list:

(1) Molinaro, A.; Holst, O.; Di Lorenzo, F.; Callaghan, M.; Nurisso, A.; D'Errico, G.; Zamyatina, A.; Peri, F.; Berisio, R.; Jerala, R.; Jimenez-Barbero, J.; Silipo, A.; Martin-Santamaria, S. *Chem. Eur. J* **2015,** 21, 500-519.
